# New and Efficient Implementation of CC3

**DOI:** 10.1021/acs.jctc.0c00686

**Published:** 2020-12-02

**Authors:** Alexander C. Paul, Rolf H. Myhre, Henrik Koch

**Affiliations:** † Department of Chemistry, Norwegian University of Science and Technology, NTNU, 7491 Trondheim, Norway; ‡ Scuola Normale Superiore, Piazza dei Cavaleri 7, 56126 Pisa, Italy

## Abstract

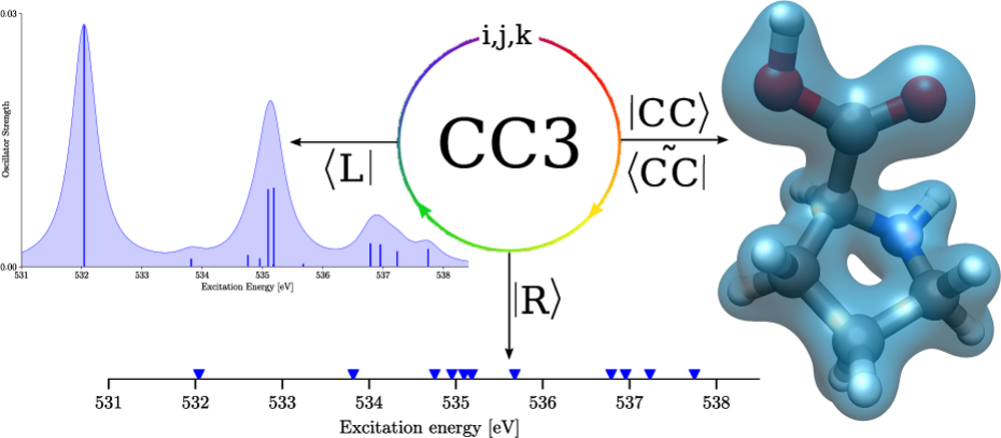

We present a new and efficient implementation of the closed shell coupled cluster singles and doubles with perturbative triples method (CC3) in the electronic structure program *e*^*T*^. Asymptotically, a ground state calculation has an iterative cost of 4*n*_V_^4^*n*_O_^3^ floating point operations (FLOP), where *n*_V_ and *n*_O_ are the number of virtual and occupied orbitals, respectively. The Jacobian and transpose Jacobian transformations, required to iteratively solve for excitation energies and transition moments, both require 8*n*_V_^4^*n*_O_^3^ FLOP. We have also implemented equation of motion (EOM) transition moments for CC3. The EOM transition densities require recalculation of triples amplitudes, as *n*_V_^3^*n*_O_^3^ tensors are not stored in memory. This results in a noniterative computational cost of 10*n*_V_^4^*n*_O_^3^ FLOP for the ground state density and 26*n*_V_^4^*n*_O_^3^ FLOP per state for the transition densities. The code is compared to the CC3 implementations in CFOUR, DALTON, and PSI4. We demonstrate the capabilities of our implementation by calculating valence and core excited states of l-proline.

## Introduction

X-ray spectroscopies such as near-edge X-ray absorption fine structure (NEXAFS) can provide detailed insight into the electronic structure of molecules and their local environment.^1,2^ With the new facilities at the European XFEL and LCLS2 at SLAC, the number of high-resolution spectroscopic experiments is increasing. Accurate modeling is a great aid when interpreting spectroscopic data, providing new insights into the underlying chemistry. However, modeling the high energy excitations measured using X-ray spectroscopy is challenging because they typically generate a core hole which in turn results in a large contraction of the electron density. To accurately describe this contraction, one has to include either triple excitations or explicit excited state orbital relaxation in the wave function description.^3−6^

Coupled cluster theory is the preferred model when calculating spectroscopic properties for molecules, combining high accuracy and correct scaling with system size in the coupled cluster response theory (CCRT) formulation.^7−9^ Coupled cluster singles and doubles (CCSD) is the most widely used variant of coupled cluster because of its high accuracy and relatively feasible computational scaling of , where *n*_V_ is the number of virtual and *n*_O_ is the number of occupied orbitals. Nevertheless, for some properties like core excitation energies, CCSD can deviate by several electron volts from experimental values. These deviations are reduced by an order of magnitude if triples are included in the description of the wave function.^4,10^ However, coupled cluster singles, doubles, and triples (CCSDT) is usually unfeasible because of the *n*_V_^3^*n*_O_^3^ memory requirement and  computational cost. Approximating the triples amplitudes can reduce the computational cost to 4*n*_V_^4^*n*_O_^3^ floating point operations (FLOP) and the required memory to *n*_V_^2^*n*_O_^2^. Note that this is twice the scaling usually reported in the literature because a matrix–matrix multiplication involves an addition and a multiplication.

Approximate triples models are typically categorized as noniterative and iterative models. For the noniterative models, a triples energy correction is computed after solving the CCSD equations. The terms included in the energy correction are usually determined based on a many-body perturbation theory (MBPT) like expansion of the energy.^11−14^ However, CCSD(T), by far the most popular of these methods, does not follow a strict MBPT expansion of the energy. For CCSD(T), the energy is expanded consistently to fourth order, and one additional fifth order term is added.^15^ It was later shown that CCSD(T) can be viewed as an MBPT-like expansion from the CCSD wave function.^16^ Similar approaches have also been proposed for excitation energies.^17,18^ A related method is the ΛCCSD(T) method where the parameters of the left wave function are included in the MBPT expansion.^19,20^ The completely renormalized CCSD(T) method, intended for multireference states, has also been extended to excited states.^21^ Other models include CCSDR3^22^ and EOM CCSD*,^23,24^ where a triples correction is added to the CCSD excitation energies. The iterative methods are generally more computationally expensive than the noniterative methods, but they are usually more accurate. The CCSDT-*n* models^25^ and CC3^26,27^ are the most well known of these methods. The two models have the same computational cost, but CC3 is more accurate because of the full inclusion of single excitation amplitudes.^28^ Recently, CC3 ground and excited states were combined with the pair natural orbital approximation in order to extend the model to larger systems.^29^ For a more extensive discussion of approximate triples methods and their accuracy, see refs. (30−35).

Because of the high computational cost, an efficient CC3 implementation is required for larger molecules.^26,27^ In this paper, we present an implementation of CC3 ground and excited states, as well as equation of motion (EOM)^36^ transition moments. Although the EOM formalism has been shown to be less accurate than CCRT for transition moments,^8^ the differences are believed to be small for high-level methods like CC3.^37,38^ The current implementation has an iterative cost of 4*n*_V_^4^*n*_O_^3^ FLOP for the ground state and 8*n*_V_^4^*n*_O_^3^ FLOP for excited states. For comparison, the old CC3 excited states implementation in DALTON has an iterative cost of 30*n*_V_^4^*n*_O_^3^ FLOP, and the new implementation in DALTON requires 10*n*_V_^4^*n*_O_^3^ FLOP per iteration.^27^ Note that it is erroneously stated in the literature that the minimal computational cost is 10*n*_V_^4^*n*_O_^3^ FLOP per iteration.^39^ For core excited states, we use the core valence separation (CVS) approximation.^40−42^ This reduces the iterative computational cost to 8*n*_V_^4^*n*_O_^2^ FLOP for excitation energies; however, the computational cost of the ground state calculation remains unchanged.

## Theory

In this section, we will derive the equations for closed shell CC3 within the EOM formalism. Note that almost all the equations in this section are equally applicable in the open shell case by changing the definitions of the Hamiltonian and the one-electron operator. Consider the coupled cluster wave function.
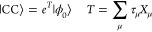
1Here, |ϕ_0_⟩ is a canonical reference Slater determinant, usually the Hartree–Fock wave function, and *T* is the cluster operator with μ labeling unique excited determinants. The excitation operator, *X*_μ_, maps the reference, |ϕ_0_⟩, into determinant |μ⟩, and τ_μ_ is the corresponding parameter, referred to as an amplitude. In the closed shell case, *X*_μ_ is defined as a string of standard singlet excitation operators, *E*_*ai*_. For example, a double excitation operator is given in eq 2.
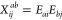
2We use the standard, notation, where the indices *i*, *j*, *k*... refer to occupied, *a*, *b*, *c*... to virtual, and *p*, *q*, *r*... to general orbitals. We will work in a biorthonormal basis and define a contravariant excitation operator, *X̃*_μ_, so that the left space is spanned by determinants biorthonormal to the right.^43^
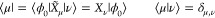
3

In order to obtain the ground state energy, we introduce a biorthonormal parametrization for the left state.
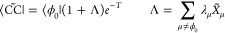
4Inserting these expressions into the Schrödinger equation, we obtain the coupled cluster Lagrangian.
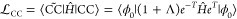
5*Ĥ* is the electronic Hamiltonian.^43^
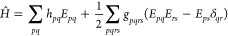
6The equations for full configuration interaction are recovered from this Lagrangian if the excitation space is not truncated. The biorthonormal left side then becomes equivalent to the conjugate of the right side up to a normalization factor.

Determining the stationary points of  results in the equations for the parameters **τ** and **λ**. The derivatives with respect to **λ** give the familiar coupled cluster projection equations for the amplitudes, and the derivatives with respect to **τ** give the equations for **λ**. In practice, *T* and Λ are truncated at some excitation level with respect to the reference determinant. For example, the CCSDT cluster operators are defined as the sum of the singles, doubles, and triples cluster operators.
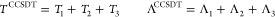
7

The exp(*T*_1_) operator can be viewed as a biorthogonal orbital transformation, and we employ the *T*_1_-transformed Hamiltonian throughout.
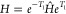
8Note that we do not use the standard notation to avoid over dressing of the operators. The equations for CCSDT then become those of CCDT. Inserting these definitions into , we get the CCSDT Lagrangian.
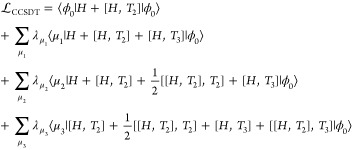
9The last two commutator terms of eq 9 make the cost of the full CCSDT model scale as . To reduce the cost, we use a perturbation scheme,^26,27^ where the transformed Hamiltonian is divided into an effective one-particle operator and a fluctuation potential, similar to MBPT.^11,12^
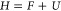
10The operators are assigned orders, as summarized in Table 1.
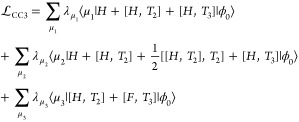
11

**Table 1:** Perturbation Orders for CC3

order	0	1	2
Hamiltonian^a^	*F*_oo_, *F*_vv_	*F*_vo_, *F*_ov_, *U*	
ground state^b^	Λ_μ_1__, *T*_μ_1__	Λ_μ_2__, *T*_μ_2__	Λ_μ_3__, *T*_μ_2__
EOM^c^	*r*, *l*, *L*_μ_1__, *R*_μ_1__	*L*_μ_2__, *R*_μ_2__	*L*_μ_3__, *R*_μ_3__

a *F*_oo_ and *F*_vv_ refer to the diagonal blocks of the Fock matrix, while *F*_vo_ and *F*_ov_ refer to the off-diagonal blocks.

b *T* and Λ refer to ground state parameters.

c *r*, *l*, *L*, and *R* refer to EOM parameters.

The CC3 Lagrangian, as shown in eq 11, is obtained by discarding terms from the CCSDT Lagrangian, that are of fifth order in the perturbation or higher, assuming a canonical basis. The singles amplitudes, both in Λ_1_ and *T*_1_, are considered to be zeroth order in the perturbation, as they are viewed as approximate orbital transformation parameters,^44,45^ in contrast to MBPT, where the first contribution of the single excitations appears in second order.

In coupled cluster theory, excitation energies and other spectroscopic properties are usually computed using either CCRT or the EOM formalism. In CCRT, time-dependent expectation values of molecular properties are expanded in orders of a frequency-dependent perturbation. The frequency-dependent expansion terms are referred to as response functions and excitation energies, and transition moments are determined from the poles and residues of the linear response function. In EOM theory, the starting point is a CI-like parametrization for the excited states. The eigenvalue problem for the Hamiltonian in this basis gives excited states and excitation energies. CCRT and EOM give the same expressions for the excitation energies; however, the transition moments differ.

To solve the EOM equations, the similarity-transformed Hamiltonian is projected onto the reference and the truncated excitation space, resulting in the Hamiltonian matrix.
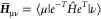
12This matrix is not symmetric; hence, the left and right eigenvectors will not be Hermitian conjugates, but they will be biorthonormal.
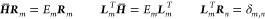
13

We introduce the convenient notation
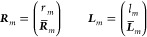
14where *l*_*m*_ and *r*_*m*_ refer to the first element of the vectors, and ***L̅***_*m*_ and ***R̅***_*m*_ refer to the rest. The vectors ***L***_*m*_ and ***R***_*m*_ correspond to the operators *L*_*m*_ and *R*_*m*_, which have a similar form as Λ and *T*, but also include reference contributions.

The EOM right and left excited states are given by eq 15 and eq 16, respectively.
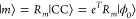
15
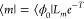
16Because the **τ** amplitudes are solutions to the coupled cluster ground state equations, the first column of ***H̅*** is zero, except for the first element which equals the ground state energy, *E*_0_, and the eigenvalues of ***H̅*** correspond to the energies of the EOM states.
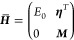
17

In the following, the index *m* will refer to states other than the ground state, which is denoted by 0. From the structure of the Hamiltonian matrix, we see that the vector ***R***_0_, with the elements *r*_0_ = 1 and ***R̅***_0_ = **0**, corresponds to the ground state. For the right excited states, ***R̅***_*m*_ must be an eigenvector of ***M*** with eigenvalue *E*_*m*_. Similarly, for the left excited states, *l*_*m*_ = 0 and ***L̅***_*m*_ has to be a left eigenvector of ***M*** because of the biorthonormality with ***R***_0_ and ***R***_*m*_. The left ground state, ***L***_0_, has the component *l*_0_ = 1, and the vector ***L̅***_0_ is obtained from eq 18, where ***I*** is the identity matrix.
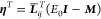
18Finally,  to ensure biorthogonality between ***R***_*m*_ and ***L***_0_. The matrix ***J*** = (***M*** – *E*_0_***I***) is the derivative of the Lagrangian with respect to **τ** and **λ** and is called the Jacobian.
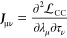
19As required, the equation for ***L***_0_ is the same as for Λ. The CCSDT Jacobian is given in eq 20, where T_2+3_ is shorthand notation for T_2_ +T_3_.
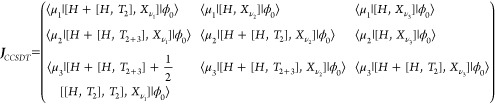
20The CCSDT **η** vector is given by eq 21.
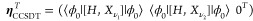
21These expressions are written in commutator form which requires that the projection equations for *T* are satisfied.

For EOM CC3, we introduce a perturbation expansion. Our starting point is the expression for the energy of the EOM states.
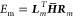
22We assign the same perturbation orders to ***L*** and ***R*** as to *T* and Λ; see Table 1. As CC3 does not satisfy the projection equations, the first column of ***H̅*** will not be zero after the first element. However, discarding the terms that are fifth order or higher, we are left with the expressions for the CC3 ground state residuals which are zero. In order to derive the correct CC3 Jacobian, known from CCRT,^46^ we discard terms from the CCSDT Jacobian in commutator form using perturbation theory.
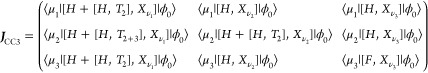
23

To obtain EOM biorthogonal expectation values, the biorthogonal states are inserted into the expressions for the CI expectation values. For a given one-electron operator, *A* = ∑_*pq*_*A*_*pq*_*E*_*pq*_, the biorthogonal expectation values are expressed in terms of left and right transition density matrices,^47,48^***D̃***^n,*m*^ and ***D***^*n*,*m*^.
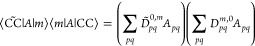
24The elements of the right transition density are defined in eq 25,
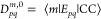
25while the elements of ***D̃***^0,*m*^ are given in eq 26,
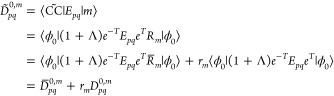
26where ***D***^0,0^ is the ground state density.

## Implementation

The closed shell CC3 ground state, singlet excitation energies, and EOM transition moments have been implemented in the *e*^*T*^ program.^49^ The core part of the algorithms is a triple loop over the occupied indices *i* ≥ *j* ≥ *k*, as proposed for CCSD(T) by Rendell et al.,^50^ and has been used in several other implementations.^27,51,52^ Within the triple loop, we first construct the triples amplitudes for a given set of {*i*, *j*, *k*} and contract them with integrals to obtain the contribution to the resulting vector. By restricting the loop indices and exploiting the permutational symmetry.
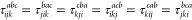
27The computational cost of constructing the triples amplitudes is reduced by a factor of six. An outline of the algorithm to construct the triples contribution to the ground state residual, **Ω**, is given in Algorithm 1. Integrals in *T*_1_-transformed basis are denoted by *g*_*pqrs*_. The equation for the triples amplitudes includes a permutation operator, defined in eq 28,
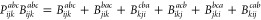
28and the orbital energy difference, defined in eq 29,
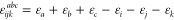
29where ε_*p*_ is the energy of orbital *p*. To recover all contributions to the **Ω** vector from the restricted loops, all unique permutations of *i*, *j*, *k* have to be considered. This results in six terms when all the occupied indices are unique and three terms when two occupied indices are equal. If all three occupied indices are identical, there is no contribution, as this corresponds to a triple excitation from a single orbital. In order to avoid reading two-electron integrals from file inside the loop, the program checks if all integrals can be kept in memory; otherwise, they are read in batches of *i*, *j*, *k* in additional outer loops. To minimize reordering inside the loop and ensure efficient matrix contractions, the integrals are reordered and written to disk before entering the loop.

Asymptotically, reordering of the amplitudes or making linear combinations of them scale as *n*_V_^3^*n*_O_^3^. However, these operations are typically memory-bound. For example, reordering the amplitudes from 123 to 312 ordering took 57 seconds, while the fastest *n*_V_^4^*n*_O_^3^ matrix multiplication took 240 seconds for a system with 431 virtual and 29 occupied orbitals. The calculation was run on a node with two Intel Xeon-Gold 6138 2.0 GHz CPUs with 20 cores each and 320 GB of memory. Reordering times are highly dependent on hardware and compiler, but it is clear that they are significant and constructing linear combinations is even more time-consuming. By constructing contravariant triples amplitudes given by eq 30, no additional linear combinations are required to construct the contravariant residual Ω̃.
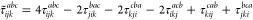
30This residual can then be transformed back to the covariant residual outside the loop.
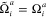
31
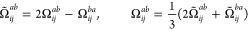
32

For systems with spatial symmetry, considerable savings could be achieved by taking symmetry into account, both in computational cost and memory. However, this results in greatly increased complexity of the code, and spatial symmetry is most relevant for small molecular systems. Consequently, it is not exploited in our implementation.

For excited state calculations, we may reduce the iterative cost from 10*n*_V_^4^*n*_O_^3^ to 8*n*_V_^4^*n*_O_^3^ FLOP by constructing **τ**_3_-dependent intermediates before entering the iterative loop. This is carried out in a preparation routine outlined in Algorithm 2. The same intermediates are used in the algorithms for both ***L*** and ***R***. Nevertheless, we still have to construct the **τ**_3_ amplitudes in each iteration; see Supporting Information. In theory, it would be possible to construct an intermediate of size *n*_V_^3^*n*_O_^3^ for this term as well, reducing the iterative computational cost to 6*n*_V_^4^*n*_O_^3^ FLOP. However, this intermediate would cost 2*n*_V_^4^*n*_O_^4^ FLOP to construct.
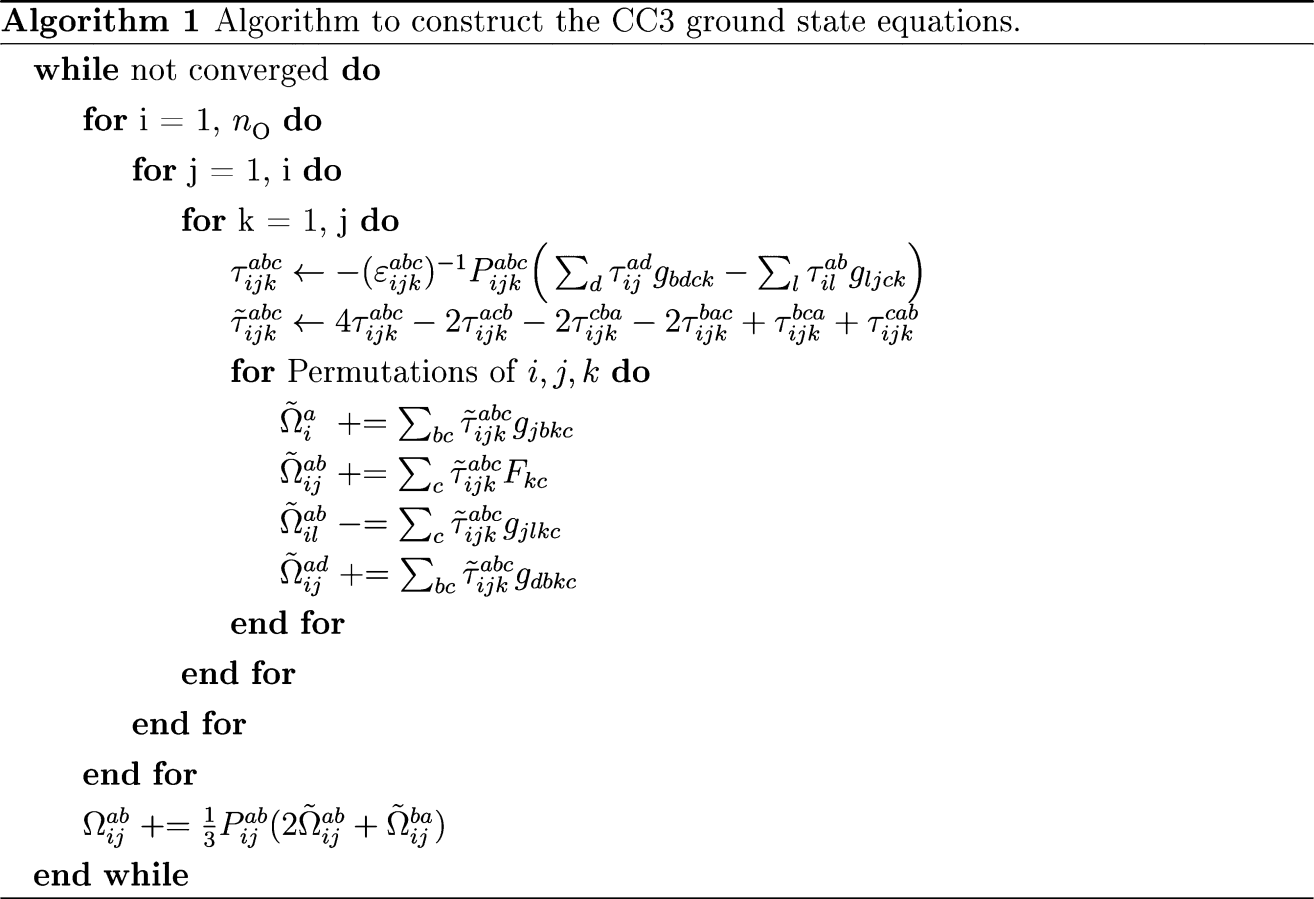


The algorithm for the Jacobian transformation of a trial vector, see Supporting Information, resembles the algorithm for the ground state, but it is separated into two loops. In the first, **τ**_3_ is constructed and contracted with an ***R***_1_-dependent intermediate. In the second loop, the routine used to construct **τ**_3_ is used again, but called twice with different input tensors to construct ***R***_3_. The excitation vector is then transformed to a contravariant form and contracted with the same integrals as the ground state to construct the excited state residual vector.

The algorithm for the transpose Jacobian transformation is similar to the right transformation. First, the **τ**_3_ amplitudes are computed and contracted in a separate loop over *i*, *j*, *k* before the main loop, where the contribution of the ***L***_3_ amplitudes is calculated. The contributions to the transpose Jacobian transform should be constructed from the contravariant form of ***L***_3_. However, constructing the contravariant form directly is complicated and requires several expensive linear combinations. The covariant form, on the other hand, can be constructed using contractions similar to those required for **τ**_3_ and six outer products, avoiding any linear combinations. The contravariant form is then obtained using eq 30. A complication for the transpose transformation is that it requires the construction of intermediates inside the *i*, *j*, *k* loop. One of these intermediates requires *n*_V_^3^*n*_O_ memory, and we have to add batching functionality, writing, and reading the intermediate from file for each batch. To avoid construction of the full *n*_V_^4^ integrals, the intermediates are contracted directly with Cholesky vectors outside the *i*, *j*, *k* loop. Asymptotically, the computational cost is 4*n*_V_^4^*n*_O_^3^ FLOP, the same as for the right transformation.
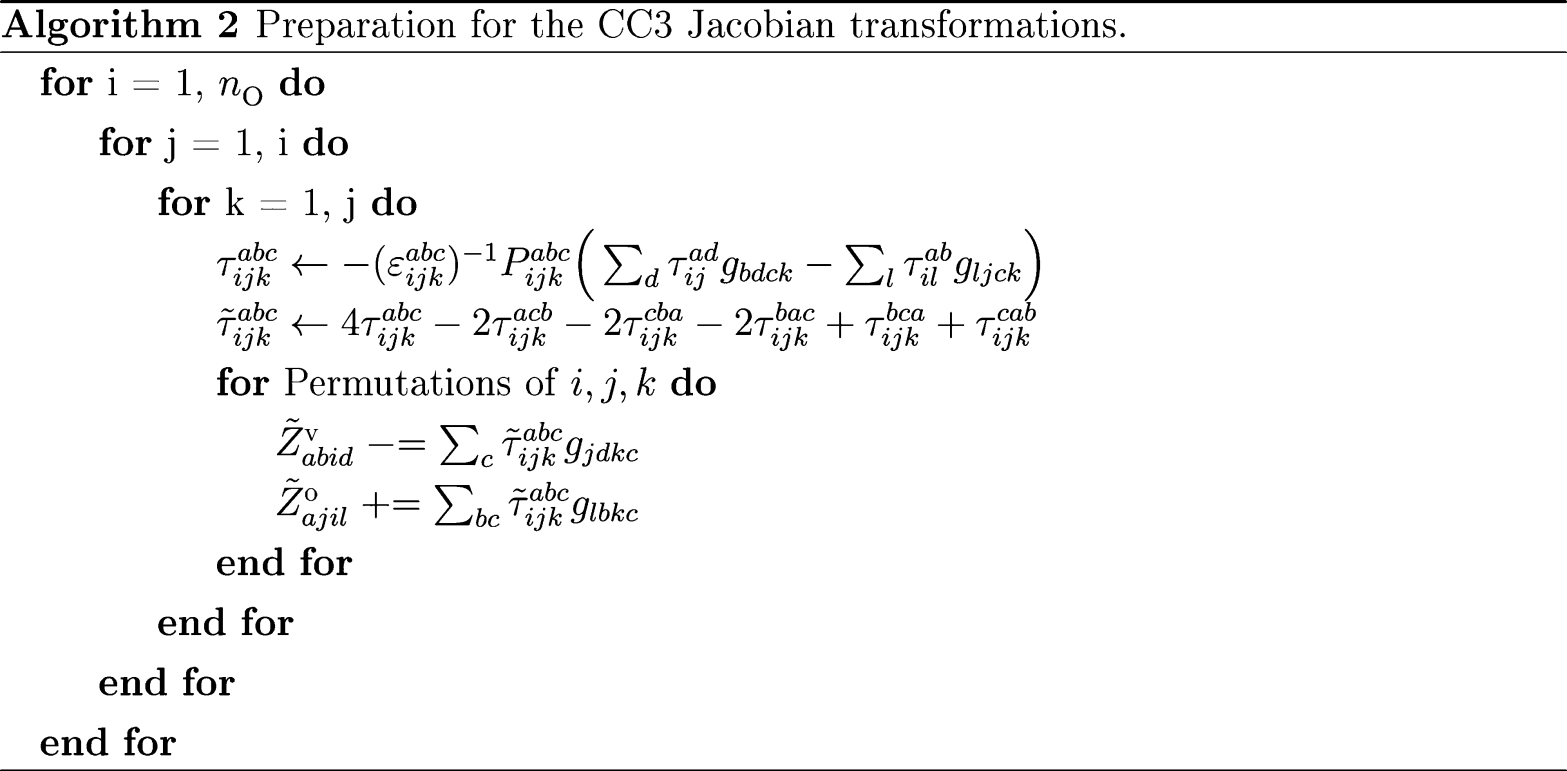


In Algorithm 3, we show how to compute the ***L***_3_ contributions to ***D***^*m*,0^; see eq 25. The same algorithm can be used to compute the ground state density, ***D***^0,0^, by inserting **Λ**_3_ instead of ***L***_3_. For ***D̃***^0,*m*^, several intermediates from the ground state density, as well as the ground state density itself, are reused, see the Supporting Information.
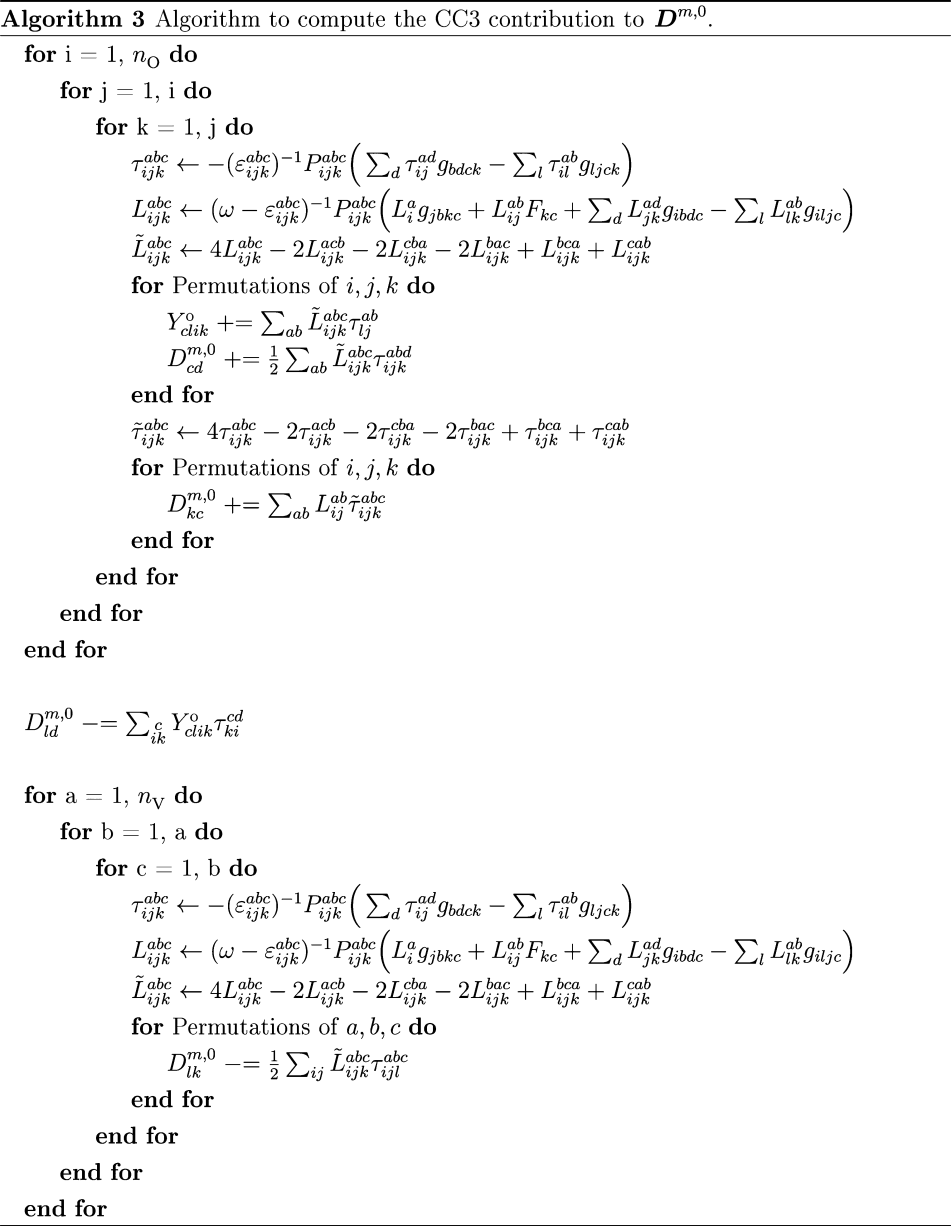


The main difference between Algorithm 3 and the algorithm for the Jacobian transformations is the additional triple loop over the virtual indices. This loop is required because of the occupied–occupied block of the density matrix that has contributions from two triples tensors with different occupied indices. Therefore, it is not possible to use the previous scheme of holding only triples amplitudes for a given *i*, *j*, *k*. In a CC3 calculation, the number of virtual orbitals is much larger than the number of occupied orbitals when a reasonable basis set is used. Therefore, the BLAS^53,54^ routines do not parallelize well, and the serial loop over the virtual indices would be inefficient. To circumvent this, the loops over the virtual indices were parallelized using OpenMP.^55^ The triples tensors have to be constructed once for fixed occupied and once for fixed virtual indices, and the computational cost of constructing the CC3 transition densities increases to 13*n*_V_^4^*n*_O_^3^ per state. Nevertheless, the construction of the densities constitutes only a small fraction of the time compared to the iterative solution of the excited state equations.

## Applications

To demonstrate the performance of the code, we have calculated the two lowest CC3 singlet valence excited states of acetamide using aug-cc-pVDZ^56^ with *e*^*T*^, PSI4,^57^ CFOUR,^46,58,59^ and the two implementations in DALTON.^39,60,61^ The timing data and the number of iterations for converging the ground state and both excited states are summarized in Table 2. When running CFOUR, the oldest DALTON implementation and PSI4, the *C*_*S*_ symmetry of acetamide has been exploited. For comparison, CFOUR was also run without symmetry. The threshold for the convergence of the ground state residual was 10^–6^, while we used a threshold of 10^–4^ for the excited states. With PSI4, both the ground and excited state residuals were converged to 10^–4^, which is why only eight iterations were needed to converge **Ω**. The differences in the convergence of the excited state equations are due to the different start guesses the programs use. While PSI4 first converges the CCSD equations and restarts CC3 from CCSD, the other programs use orbital energy differences as default start guesses. Note that all these programs can restart from the CCSD solution. As the lowest excited state is not dominated by the lowest orbital energy difference, a specific start guess had to be chosen to obtain the lowest root with CFOUR. This start guess improved the convergence behavior of CFOUR significantly. To remove the dependence on the number of iterations, we report timings per iteration which are dominated by the time spent computing the CC3 contributions. However, PSI4 does not report timings per iteration to converge the ground state equations and CFOUR does not report timings per iteration for converging the excited state equations. Therefore, the total time spent solving for the ground state and the excited states, respectively, was divided by the number of iterations. Even though the reported timings might not compare entirely identical steps in the codes, Table 2 clearly shows the efficiency of the CC3 code in *e*^*T*^.

**Table 2:** Comparison of Ground State and Excited State Calculations using CFOUR, DALTON, *e*^*T*^, and PSI4^a^

	ground state		excited states		total
	wall time [s]	*n*_Iter_^b^	wall time [s]	*n*_Iter_^b^	wall time [min]
*e*^*T*^	16	13	28	65	34
DALTON new	47	13	97	62	129
CFOUR sym	150	13	320	38	240
CFOUR no sym	330	13	685	34	468
DALTON old	267	13	767	71	971
PSI4	404	8	1187	49	1040

a The calculations were performed on one node with four Intel Xeon Gold 6130 CPU with 16 cores each using 40 cores and using a total of 180 GB shared memory.

b *n*_Iter_ specifies the number of iterations to converge the respective states.

To demonstrate the capabilities of the code, we have calculated singlet valence and core excitation energies and EOM oscillator strengths for the amino acid l-proline (C_5_H_9_NO_2_).^62^ One valence excitation energy was calculated at the CCSD/aug-cc-pVTZ and CC3/aug-cc-pVTZ levels of theory using the frozen core approximation, resulting in 23 occupied and 544 virtual orbitals.^56^

Table 3 shows the excitation energy and oscillator strength for the lowest valence excited state at the CCSD and CC3 level. The excitation vector has 96% singles contribution, and the excitation energies differ by about 0.11 eV. In Table 4, we report the averaged time per routine call as well as an estimate for the computational efficiency and the number of routine calls. For the ground state, for example, *n*_calls_ specifies the number of times the ground state residual vector is computed. The efficiency is defined as the observed FLOP per second (FLOPS) divided by the theoretical maximum number of FLOPS. For the CPUs used for this calculation, with two Intel Xeon Gold 6152 processors, the theoretical maximum is given by eq 33.^63^
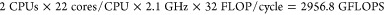
33When calculating the number of FLOP, we only count the dominant matrix–matrix multiplications with a FLOP cost of 2*n*_V_^4^*n*_O_^3^. This will be an undercount of the total FLOP, but should give a ballpark estimate. Note that the CPUs have turbo boost technology, giving a maximum theoretical frequency of 3.7 GHz when one core is active and 2.8 GHz when 22 cores are active. For the highly efficient BLAS routines used for the matrix multiplications, however, the actual frequency is likely to be close to the base frequency of 2.1 GHz.

**Table 3:** Proline Excitation Energy and Oscillator Strength for the Lowest Singlet Valence Excitation at the CCSD and CC3 Levels of Theory

CCSD		CC3	
ω [eV]	*f* × 100	ω [eV]	*f* × 100
5.830	0.0775	5.72	0.0661

**Table 4:** Timings for the Different Parts of the Calculation of One Valence Excited State with Oscillator Strengths in l-Proline at the CC3 Level of Theory

contributions	wall time [min]^a^	efficiency [%]	*n*_calls_^b^
ground state	163	14.7	10
prepare for multipliers	169	14.2	1
multipliers	347	13.8	11
prepare for Jacobian	147	16.3	1
right excited states	281	17.1	26
prepare for Jacobian	160	15.0	1
left excited states	341	14.1	28
***D***^0,0^	379	15.8	1
***D***^*m*,0^	382	15.7	1
***D̃***^0,*m*^	530	15.9	1

a Timings have been averaged over the number of routine calls. The calculations were performed on one node with two Intel Xeon Gold 6152 processors with 22 cores each and using a total of 700 GB shared memory.

b *n*_calls_ specifies the number of calls to the subroutines constructing the respective quantity.

From Table 4, we observe that one iteration of the multiplier equations is approximately twice as expensive as one iteration for the ground state. The transpose Jacobian transformation, which is required for the multipliers, costs 8*n*_V_^4^*n*_O_^3^ FLOP compared to 4*n*_V_^4^*n*_O_^3^ FLOP for the ground state. The timings to obtain left excited states are roughly the same as the timings to solve for the multipliers because a trial vector is transformed by the transpose of the Jacobian. Note that the timings in Table 4 were obtained with an older version of the code that required the construction of the full *n*_V_^4^ integrals for the left vectors and did not exploit the covariant–contravariant transformations. In the preparation routines, the intermediates used in the Jacobian transformations are computed, as shown in Algorithm 2. The preparation is as expensive as one iteration for the ground state, but we save 2*n*_V_^4^*n*_O_^3^ FLOP per Jacobian transformation. The ground state density and ***D***^*m*,0^ are calculated using the same routines and the computational cost is the same. The CC3 contribution to ***D̃***^0,*m*^ requires **τ**_3_, **λ**_3_, and ***R***_3_. In addition, ***R***_3_ is approximately twice as expensive to compute as **τ**_3_, so ***D̃***^0,*m*^ is considerably more expensive than ***D***^*m*,0^.

**Figure 1:**
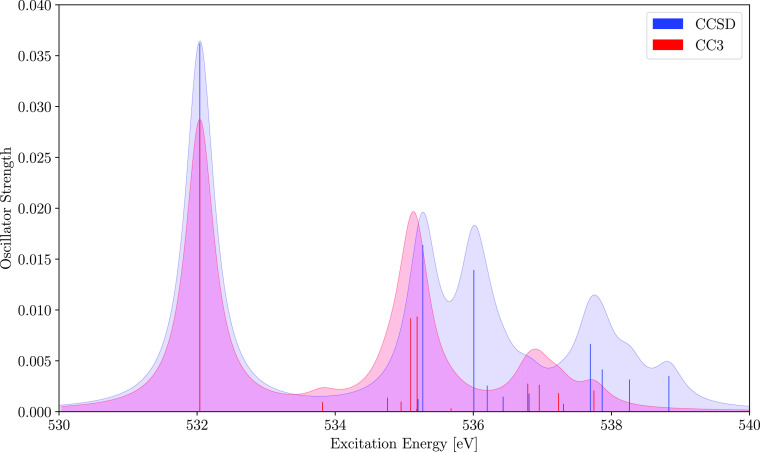
Core excitation spectrum of the oxygen atoms of l-proline computed with CC3 (red) and CCSD (blue). The peaks were broadened using a Lorentzian line shape and a width of 0.5 eV. The CCSD spectrum is shifted by −1.9 eV to match the first peak of the CC3 spectrum.

We have also calculated six core excited states for each of the oxygen atoms using CVS. The aug-cc-pCVTZ basis set was used on the oxygen atom that was excited and aug-cc-pVDZ for the rest of the molecule (31 occupied and 270 virtual orbitals).^56,64^ In Table 5, we show the results for core excitations from the carbonyl oxygen of l-proline. Because of the better description of relaxation effects by the inclusion of triple excitations, the excitation energies obtained with CC3 are up to 3 eV lower than the corresponding CCSD excitation energies. The same trends are observed for core excitations from the hydroxyl oxygen, as shown in Table 6. The CC3 oscillator strengths are between 16 and 60% lower than the values obtained with CCSD. In Figure 1, we show NEXAFS spectra computed with EOM CCSD and EOM CC3. Despite shifting the CCSD spectrum by −1.9 eV, the two spectra show significant differences. From the CCSD spectrum one would expect two peaks between 535 and 536 eV, and the peak at 534 eV is not present in the shifted CCSD plot. The calculated CC3 excitation energies are in good agreement with experimental data reported by Plekan et al. in ref (65). The authors measured the first excitation from the carbonyl oxygen at 532.2 eV and a broad peak from the hydroxyl oxygen at 535.4 eV, consistent with the first two calculated CC3 excitation energies. Note that taking relativistic effects into account will increase the excitation energies by about 0.38 eV, while increasing the basis set would lower them slightly.^10,66^

**Table 5:** Proline Excitation Energies and Oscillator Strengths for Core Excitations From the Carbonyl Oxygen at the CCSD and CC3 Levels of Theory

CCSD		CC3	
ω [eV]	*f* × 100	ω [eV]	*f* × 100
533.943	3.6218	532.040	2.8539
537.103	0.1238	533.817	0.0953
538.104	0.2566	534.756	0.1377
538.335	0.1477	534.953	0.0986
538.710	0.1779	535.179	0.0262
539.207	0.0761	535.677	0.0340

**Table 6:** Proline Excitation Energies and Oscillator Strengths for Core Excitations from the Hydroxyl Oxygen at the CCSD and CC3 Levels of Theory

CCSD		CC3	
ω [eV]	*f* × 100	ω [eV]	*f* × 100
537.172	1.6373	535.093	0.9192
537.911	1.3923	535.186	0.9351
539.598	0.6640	536.789	0.2758
539.770	0.4145	536.955	0.2643
540.165	0.3164	537.235	0.1824
540.736	0.3508	537.747	0.2088

Timings for the calculations of the core excited states are reported in Table 7 for excitations from the carbonyl oxygen. The timings for the core excitations from the hydroxyl oxygen are not reported because they are almost identical. Compared to the valence excited state calculation, the timings for the ground state and the multipliers are reduced because of the use of smaller basis sets. The CVS approximation reduces the computational cost of the Jacobian transformations from 8*n*_V_^4^*n*_O_^3^ to 8*n*_V_^4^*n*_O_^2^ FLOP.^35,67^ Therefore, one iteration is 6 times faster than a ground state iteration. These savings are achieved by cycling the triple loop over the occupied indices when none of the indices correspond to the core orbitals of interest. Similar savings can be achieved during the construction of the transition densities. However, in the present implementation, only the triple loop over the occupied indices can be cycled. The efficiency is improved compared to the valence excitation calculation, as the contravariant code was used for this calculation.

**Table 7:** Timings for the Different Parts of the Calculation of Six Core Excited States (Located at the Carbonyl Oxygen) with Oscillator Strengths for l-Proline at the CC3 Level of Theory

contributions	wall time [min]^a^	efficiency [%]	*n*_calls_^b^
ground state	19	22.2	12
prepare for multipliers	17	24.8	1
multipliers	31	26.4	14
prepare for Jacobian	16	25.2	1
right excited states	3	7.7	290
left excited states	3	8.4	315
***D***^0,0^	50	10.2	1
***D***^*m*,0^	23	8.8	6
***D̃***^0,*m*^	45	6.9	6

a Timings have been averaged over the number of routine calls. The calculations were performed on nodes with two Intel Xeon Gold 6138 processors with 20 cores each and using a total of 370 GB shared memory.

b *n*_calls_ specifies the number of calls to the subroutines constructing the respective quantities.

In Table 8, we present timings from calculations on furan with the aug-cc-pVDZ basis set, using 1, 5, 10, 20, and 40 threads. We calculated the transition moments from the ground state to the first excited state, which requires solving for **τ**, **λ**, ***R***, and ***L***. We also report speedups relative to the single thread calculation. Increasing the number of threads from 1 to 40 reduces the total wall time by approximately a factor of 15. Because of dynamic overclocking, the theoretical maximum frequency for the single threaded case is 3.7 GHz, while it is 2.7 GHz with 20 active cores per processor and the base frequency is 2.0 GHz.^63^

**Table 8:** Timings for Calculating the EOM Transition Moment for the First Excited State of Furan in Seconds Using 1, 5, 10, 20, and 40 Threads

threads	total^a^		τ (13)^b^		λ (14)^b^		*R* (15)^b^		*L* (16)^b^	
1	35197		4231		8403		9148		9605	
5	8630	4.08	1067	3.96	2059	4.08	2259	4.05	2347	4.09
10	4612	7.63	572	7.40	1103	7.62	1199	7.63	1252	7.67
20	2841	12.39	353	11.98	691	12.16	743	12.32	763	12.58
40	2286	15.39	290	14.61	563	14.94	587	15.60	632	15.19

a The speedup compared to a single core is given next to the timing. The calculations were performed on a node with two Intel Xeon Gold 6138 2.0 GHz processors with 20 cores each and using a total of 150 GB shared memory.

b Numbers of iterations are given in parentheses.

**Figure 2:**
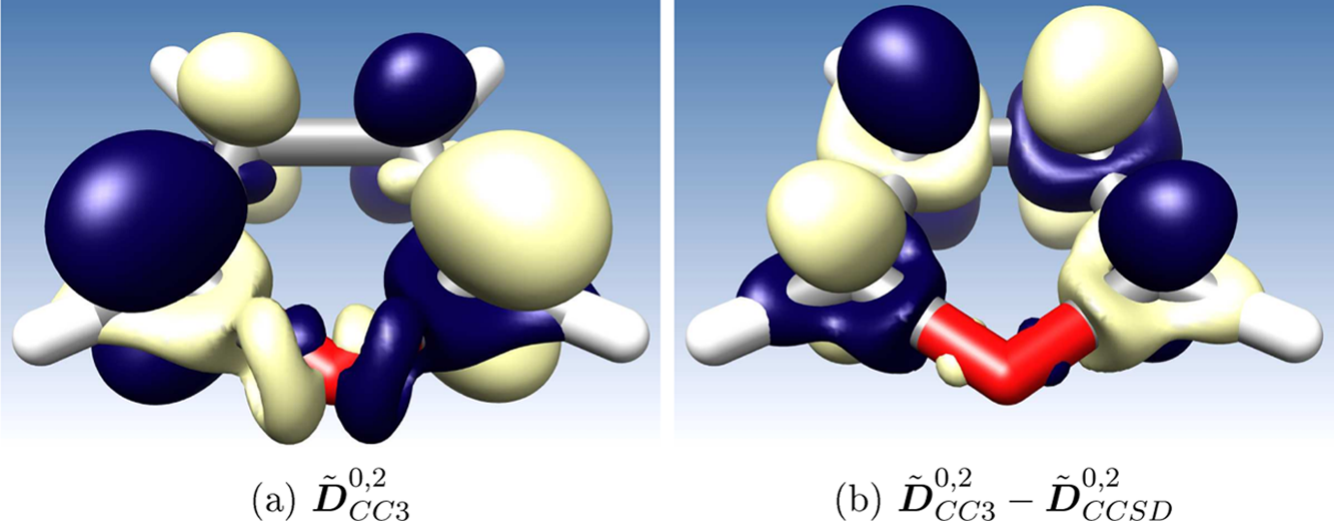
Transition densities of furan. (a) Second CC3 transition density (***D̃***^CC3^) with contour value 0.006. (b) Difference from CCSD (***D̃***_CC3_^0,2^ – ***D̃***_CCSD_^0,2^) with contour value 0.0003.

Finally, in Figure 2, we show the CC3 transition density, ***D̃***_CC3_^0,2^, as well as the difference between the CC3 and CCSD transition densities, ***D̃***_CC3_^0,2^ – ***D̃***_CCSD_^0,2^, plotted using Chimera.^68^ While the difference between the densities is small (the contour value is only 0.0003), the triples decrease the volume at the same contour value, which goes along with an increase in the double excitation character. This is reflected in a reduction of the oscillator strength from 0.181 to 0.168.

## Conclusions

In this paper, we have described an efficient implementation of the CC3 model including ground state and excited state energies as well as EOM oscillator strengths. To the best of our knowledge, the algorithm reported is the most efficient for canonical CC3 and the first implementation of EOM CC3 transition densities. The computational cost of excited states is reduced to 8*n*_V_^4^*n*_O_^3^ FLOP because of the introduction of intermediates constructed outside the iterative loop. The code is parallelized using OpenMP, and the algorithm can be extended to utilize MPI through coarrays which are included in the Fortran 2008 standard.

A possible modification of the code is to use triple loops over the virtual orbitals for the construction of the amplitudes. OpenMP parallelization will then happen at the level of the triple loops, which is already implemented for parts of the density construction. Early experimental code indicates that the efficiency of the matrix–matrix multiplications are then slightly reduced, but the overhead due to reordering almost vanishes. This is probably related to the spatial locality of the arrays in memory. Another advantage of such a scheme is that it can be adapted for graphical processing units.

Finally, the extension to the densities of excited states and the transition densities between excited states is straightforward and will be reported elsewhere.

## Supporting Information Available

Algorithms to calculate Jacobian transformations, algorithms to construct the transition densities, and geometries (PDF)
