# Supplementary material for: New and Efficient Implementation of CC3
Source: J Chem Theory Comput. 2020 Dec 2;17(1):117–26. doi: 10.1021/acs.jctc.0c00686 (PMC8016187; doi:10.1021/acs.jctc.0c00686)
Supplement: Supplementary file 1 — ct0c00686_si_001.pdf [file ct0c00686_si_001.pdf]

Supporting information for *A new and efficient implementation of CC3*

Alexander C. Paul,[†] Rolf H. Myhre,[†] and Henrik Koch[*,†,‡]

†*Department of Chemistry, Norwegian University of Science and Technology, NTNU, 7491 Trondheim, Norway*

‡*Scuola Normale Superiore, Piazza dei Cavaleri 7, 56126 Pisa, Italy*

E-mail: henrik.koch@sns.it

Supporting Information Available

Here we report equations and pseudocode for the Jacobian transformations and the transition densities. For a concise notation, we define

$$\bar{R}^{ab}_{ij} = (1 + \delta_{ij}\delta_{ab})R^{ab}_{ij}. \quad (1)$$

Algorithms to calculate Jacobian transformations

The equations and the algorithm to compute the Jacobian transformation of a trial vector $\boldsymbol{R}$ are reported in Algorithm 1. The transformed vector is denoted by $\boldsymbol{\rho}$ and the indices are chosen such that the triples amplitudes have the indices a, b, c, i, j, k. The intermediates, $\tilde{Z}^{\mathrm{v}}_{abid}$ and $\tilde{Z}^{\mathrm{o}}_{ajil}$, are constructed outside the iterative loop as discussed in the Implementation section of the paper.

The equations and the algorithm for the transpose Jacobian transformation are shown in Algorithm 2 where $\boldsymbol{\sigma}$ denotes the transformed vector.

Algorithm 1 CC3 Jacobian transformation algorithm.

while not converged **do**

$\Upsilon_{kc} \leftarrow \sum_{\substack{d\\l}} (2g_{kcld} - g_{kdlc}) R_l^d$

for i = 1, n_O **do**

for j = 1, i **do**

for k = 1, j **do**

$\tau_{ijk}^{abc} \leftarrow -(\varepsilon_{ijk}^{abc})^{-1} P_{ijk}^{abc} \Big(\sum_d \tau_{ij}^{ad} g_{bdck} - \sum_l \tau_{il}^{ab} g_{ljck} \Big)$

$\tilde{\tau}_{ijk}^{abc} \leftarrow 4\tau_{ijk}^{abc} - 2\tau_{ijk}^{acb} - 2\tau_{ijk}^{cba} - 2\tau_{ijk}^{bac} + \tau_{ijk}^{bca} + \tau_{ijk}^{cab}$

for Permutations of i, j, k **do**

$\tilde{\rho}_{ij}^{ab} \mathrel{+}= \sum_c \tilde{\tau}_{ijk}^{abc} \Upsilon_{kc}$

end for

end for

end for

end for

$\Upsilon_{bdck} \leftarrow \sum_e R_k^e g_{bdce} - \sum_m \left(R_m^b g_{mdck} + R_m^c g_{bdmk} \right)$

$\Upsilon_{ljck} \leftarrow \sum_e \left(R_j^e g_{leck} + R_k^e g_{ljce} \right) - \sum_m R_m^c g_{ljmk}$

for i = 1, n_O **do**

for j = 1, i **do**

for k = 1, j **do**

$$R_{ijk}^{abc} \leftarrow (\omega - \varepsilon_{ijk}^{abc})^{-1} P_{ijk}^{abc} \Big(\sum_d \bar{R}_{ij}^{ad} g_{bdck} - \sum_l \bar{R}_{il}^{ab} g_{ljck} + \sum_d \tau_{ij}^{ad} \Upsilon_{bdck} - \sum_l \tau_{il}^{ab} \Upsilon_{ljck} \Big)$$

$\tilde{R}_{ijk}^{abc} \leftarrow 4R_{ijk}^{abc} - 2R_{ijk}^{acb} - 2R_{ijk}^{cba} - 2R_{ijk}^{bac} + R_{ijk}^{bca} + R_{ijk}^{cab}$

for Permutations of i, j, k **do**

$\rho_i^a \mathrel{+}= \sum_{bc} \tilde{R}_{ijk}^{abc} g_{jbkc}$

$\tilde{\rho}_{ij}^{ab} \mathrel{+}= \sum_c \tilde{R}_{ijk}^{abc} F_{kc}$

$\tilde{\rho}_{il}^{ab} \mathrel{-}= \sum_c \tilde{R}_{ijk}^{abc} g_{jlkc}$

$\tilde{\rho}_{ij}^{ad} \mathrel{+}= \sum_{bc} \tilde{R}_{ijk}^{abc} g_{dbkc}$

end for

end for

end for

end for

$\tilde{\rho}_{il}^{ab} \mathrel{+}= \sum_d \tilde{Z}_{abid}^{\mathrm{v}} R_l^d$

$\tilde{\rho}_{ij}^{ad} \mathrel{-}= \sum_l \tilde{Z}_{ajil}^{\mathrm{o}} R_l^d$

$\rho_{ij}^{ab} \mathrel{+}= \frac{1}{3(1+\delta_{ai,bj})} P_{ij}^{ab} (2\tilde{\rho}_{ij}^{ab} + \tilde{\rho}_{ij}^{ba})$

end while

Algorithm 2 CC3 transpose Jacobian transformation algorithm.

while not converged **do**
  for i = 1, n_O **do**
    for j = 1, i **do**
      for k = 1, j **do**
        $\tau_{ijk}^{abc} \leftarrow -(\varepsilon_{ijk}^{abc})^{-1} P_{ijk}^{abc} \Big(\sum_d \tau_{ij}^{ad} g_{bdck} - \sum_l \tau_{il}^{ab} g_{ljck} \Big)$
        $\tilde{\tau}_{ijk}^{abc} \leftarrow 4\tau_{ijk}^{abc} - 2\tau_{ijk}^{acb} - 2\tau_{ijk}^{cba} - 2\tau_{ijk}^{bac} + \tau_{ijk}^{bca} + \tau_{ijk}^{cab}$
        for Permutations of i, j, k **do**
          $Z_{ai} \mathrel{+}= \sum_{bc} L_{jk}^{bc} \tilde{\tau}_{ijk}^{abc}$
        end for
      end for
    end for
  end for
  for i = 1, n_O **do**
    for j = 1, i **do**
      for k = 1, j **do**
        $L_{ijk}^{abc} \leftarrow (\omega - \varepsilon_{ijk}^{abc})^{-1} P_{ijk}^{abc} \Big(L_i^a g_{jbkc} + L_{ij}^{ab} F_{kc} + \sum_d L_{jk}^{ad} g_{ibdc} - \sum_l L_{lk}^{ab} g_{iljc} \Big)$
        $\tilde{L}_{ijk}^{abc} \leftarrow 4L_{ijk}^{abc} - 2L_{ijk}^{acb} - 2L_{ijk}^{cba} - 2L_{ijk}^{bac} + L_{ijk}^{bca} + L_{ijk}^{cab}$
        for Permutations of i, j, k **do**
          $\sigma_{ij}^{ad} \mathrel{+}= \sum_{bc} \tilde{L}_{ijk}^{abc} g_{bdck}$
          $\sigma_{il}^{ab} \mathrel{-}= \sum_{c} \tilde{L}_{ijk}^{abc} g_{ljck}$
          $Y_{bcek}^{\mathrm{v}} \mathrel{+}= \sum_{a} \tilde{L}_{ijk}^{abc} \tau_{ij}^{ae}$
          $Y_{cmjk}^{\mathrm{o}} \mathrel{+}= \sum_{ab} \tilde{L}_{ijk}^{abc} \tau_{im}^{ab}$
        end for
      end for
    end for
  end for
  $\sigma_l^d \mathrel{+}= \sum_{ai} Z_{ai}(2g_{iald} - g_{idla}) + \sum_{\substack{ab\\i}} L_{il}^{ab} \tilde{Z}_{abid}^{\mathrm{v}} - \sum_{\substack{a\\ij}} L_{ij}^{ad} \tilde{Z}_{ajil}^{\mathrm{o}}$
  $\sigma_l^d \mathrel{+}= \sum_{jkm} Y_{dmjk}^{\mathrm{o}} g_{mjlk} - \sum_{cmj} Y_{cmjl}^{\mathrm{o}} g_{mjcd} - \sum_{cmk} Y_{cmlk}^{\mathrm{o}} g_{mdck}$
  $\sigma_l^d \mathrel{+}= \sum_{bec} Y_{bcel}^{\mathrm{v}} g_{becd} - \sum_{bek} Y_{bdek}^{\mathrm{v}} g_{lkbe} - \sum_{cek} Y_{dcek}^{\mathrm{v}} g_{leck}$
  $\sigma_{ij}^{ab} \leftarrow P_{ij}^{ab} \sigma_{ij}^{ab}$
end while

Algorithms to construct the transition densities

In the following the CC3 contributions to the transition densities $\boldsymbol{D}^{m,0}$ and $\tilde{\boldsymbol{D}}^{0,m}$ are summarized. The CCSD terms can be found for example in Table II in Ref. 1.

Substituting L_μ in the equations for $\boldsymbol{D}^{m,0}$ with λ_μ results in the equations for the ground state density, $\boldsymbol{D}^{0,0}$. In this section only the algorithm for $\tilde{\boldsymbol{D}}^{0,m}$ is shown, the algorithm for $\boldsymbol{D}^{m,0}$ can be found in the main paper.

Algorithm 3 Algorithm to compute the CC3 contribution to $\tilde{\boldsymbol{D}}^{0,m}$.

for i = 1, n_O **do**
  for j = 1, i **do**
    for k = 1, j **do**
      $R^{abc}_{ijk} \leftarrow (\omega - \varepsilon^{abc}_{ijk})^{-1} P^{abc}_{ijk}\Big(\sum_d \bar{R}^{ad}_{ij} g_{bdck} - \sum_l \bar{R}^{ab}_{il} g_{ljck} + \sum_d \tau^{ad}_{ij} \Upsilon_{bdck} - \sum_l \tau^{ab}_{il} \Upsilon_{ljck}\Big)$
      $\lambda^{abc}_{ijk} \leftarrow -(\varepsilon^{abc}_{ijk})^{-1} P^{abc}_{ijk}\Big(\lambda^a_i g_{jbkc} + \lambda^{ab}_{ij} F_{kc} + \sum_d \lambda^{ad}_{jk} g_{ibdc} - \sum_l \lambda^{ab}_{lk} g_{iljc}\Big)$
      $\tilde{\lambda}^{abc}_{ijk} \leftarrow 4\lambda^{abc}_{ijk} - 2\lambda^{acb}_{ijk} - 2\lambda^{cba}_{ijk} - 2\lambda^{bac}_{ijk} + \lambda^{bca}_{ijk} + \lambda^{cab}_{ijk}$
      for Permutations of i, j, k **do**
        $\tilde{D}^{0,m}_{cd}$ += $\frac{1}{2} \sum_{ab} \tilde{\lambda}^{abc}_{ijk} R^{abd}_{ijk}$
        $\tilde{D}^{0,m}_{ck}$ += $\sum_{ab} \tilde{\lambda}^{abc}_{ijk} \bar{R}^{ab}_{ij}$
        $\tilde{\Gamma}_{bcjk}$ += $\sum_a \tilde{\lambda}^{abc}_{ijk} R^a_i$
        overlap += $\frac{1}{1+\delta_{i,j}+\delta_{j,k}} \sum_{abc} \tilde{\lambda}^{abc}_{ijk} R^{abc}_{ijk}$
      end for
    end for
  end for
end for

for a = 1, n_V **do**
  for b = 1, a **do**
    for c = 1, b **do**
      $R^{abc}_{ijk} \leftarrow (\omega - \varepsilon^{abc}_{ijk})^{-1} P^{abc}_{ijk}\Big(\sum_d \bar{R}^{ad}_{ij} g_{bdck} - \sum_l \bar{R}^{ab}_{il} g_{ljck} + \sum_d \tau^{ad}_{ij} \Upsilon_{bdck} - \sum_l \tau^{ab}_{il} \Upsilon_{ljck}\Big)$
      $\lambda^{abc}_{ijk} \leftarrow -(\varepsilon^{abc}_{ijk})^{-1} P^{abc}_{ijk}\Big(\lambda^a_i g_{jbkc} + \lambda^{ab}_{ij} F_{kc} + \sum_d \lambda^{ad}_{jk} g_{ibdc} - \sum_l \lambda^{ab}_{lk} g_{iljc}\Big)$
      $\tilde{\lambda}^{abc}_{ijk} \leftarrow 4\lambda^{abc}_{ijk} - 2\lambda^{acb}_{ijk} - 2\lambda^{cba}_{ijk} - 2\lambda^{bac}_{ijk} + \lambda^{bca}_{ijk} + \lambda^{cab}_{ijk}$
      for Permutations of a, b, c **do**
        $\tilde{D}^{0,m}_{kl}$ −= $\frac{1}{2} \sum_{ij} \tilde{\lambda}^{abc}_{ijl} R^{abc}_{ijk}$
      end for
    end for
  end for
end for
$\tilde{D}^{0,m}_{pq}$ += $\sum_{i=1}^{3} R_{\mu_i} \lambda_{\mu_i} \big(2\delta_{pq}\delta_{p,occ} - D^{0,0}_{pq}\big)$

Algorithm 4 Contributions from precalculated intermediates to $\tilde{\boldsymbol{D}}^{0,m}$.

$\tilde{D}^{0,m}_{lk} \mathrel{+}= \sum_{\substack{a\\i}} Y^{\mathrm{o}}_{alik} R^a_i \equiv -\sum_{\substack{abc\\ij}} \lambda^{abc}_{ijk} \tau^{bc}_{jl} R^a_i$

$\tilde{D}^{0,m}_{cd} \mathrel{+}= \sum_{\substack{a\\i}} Y^{\mathrm{v}}_{acdi} R^a_i \equiv \sum_{\substack{ab\\ijk}} \lambda^{abc}_{ijk} \tau^{bd}_{jk} R^a_i$

$\tilde{D}^{0,m}_{ld} \mathrel{+}= \frac{1}{2} \sum_{\substack{abc\\ijk}} \lambda^{abc}_{ijk} \tau^{abd}_{ijk} R^c_l - \frac{1}{2} \sum_{\substack{abc\\ijk}} \lambda^{abc}_{ijk} \tau^{abc}_{ijl} R^d_k$

$\tilde{D}^{0,m}_{ld} \mathrel{+}= -\sum_{\substack{ac\\i}} Y^{\mathrm{v}}_{acdi} \bar{R}^{ac}_{il} - \sum_{\substack{a\\ik}} Y^{\mathrm{o}}_{alik} \bar{R}^{ad}_{ik}$

$\Gamma_{bcjk} = \frac{1}{3}(2\tilde{\Gamma}_{bcjk} + \tilde{\Gamma}_{cbjk})$

for i = 1, n_{O} **do**

  for j = 1, i **do**

    for k = 1, j **do**

      $\tau^{abc}_{ijk} \leftarrow -(\varepsilon^{abc}_{ijk})^{-1} P^{abc}_{ijk} \left(\sum_d \tau^{ad}_{ij} g_{bdck} - \sum_l \tau^{ab}_{il} g_{ljck} \right)$

      $\tilde{\tau}^{abc}_{ijk} \leftarrow 4\tau^{abc}_{ijk} - 2\tau^{acb}_{ijk} - 2\tau^{cba}_{ijk} - 2\tau^{bac}_{ijk} + \tau^{bca}_{ijk} + \tau^{cab}_{ijk}$

      for Permutations of i, j, k **do**

        $\tilde{D}^{0,m}_{ia} \mathrel{+}= \sum_{\substack{bc\\jk}} \tilde{\tau}^{abc}_{ijk} \Gamma_{bcjk}$

      end for

    end for

  end for

end for

Geometries

Here we list the geometries of the molecules used in the calculations presented in the application section of the paper.

Table 1: Geometry of acetamide in Ångstrom obtained from Ref. 2.

Atom	x	y	z
O	0.000 000	1.229 439	0.000 000
N	1.158 967	−0.727 718	0.000 000
C	−1.267 042	−0.831 610	0.000 000
C	0.000 000	0.000 000	0.000 000
H	1.173 209	−1.735 763	0.000 000
H	2.035 841	−0.226 201	0.000 000
H	−2.121 189	−0.156 089	0.000 000
H	−1.310 647	−1.472 742	0.885 504
H	−1.310 647	−1.472 742	−0.885 504

Table 2: Geometry of L-proline in Ångstrom obtained from Ref. 3.

Atom	x	y	z
O	2.1617	−0.9509	−0.1119
O	1.5688	1.2164	−0.4953
N	−0.7807	0.9314	0.7770
C	0.0834	−0.2476	0.7251
C	−0.7235	−1.3171	−0.0031
C	−1.9888	−0.6106	−0.4752
C	−1.6499	0.8635	−0.3933
C	1.3289	0.1149	−0.0235
H	0.3625	−0.5522	1.7389
H	−0.9873	−2.1429	0.6671
H	−0.1919	−1.7411	−0.8624
H	−2.2798	−0.9157	−1.4848
H	−2.8187	−0.8485	0.2018
H	−1.1204	1.1932	−1.2945
H	−2.5424	1.4831	−0.2694
H	−0.2509	1.8003	0.8023
H	2.9837	−0.7341	−0.6014

Table 3: Geometry of furan in Ångstrom. The geometry was optimized using B3LYP with def2-TZVP basis set as implemented in Orca.[4–7]

Atom	x	y	z
O	0.000 000	0.000 000	1.128 606
C	0.000 000	0.716 455	−0.983 624
C	0.000 000	−0.716 455	−0.983 624
C	0.000 000	1.094 306	0.318 066
C	0.000 000	−1.094 306	0.318 066
H	0.000 000	1.370 213	−1.839 429
H	0.000 000	−1.370 213	−1.839 429
H	0.000 000	2.048 735	0.813 913
H	0.000 000	−2.048 735	0.813 913

References

(1) Stanton, J. F.; Bartlett, R. J. The equation of motion coupled-cluster method. A systematic biorthogonal approach to molecular excitation energies, transition probabilities, and excited state properties. *J. Chem. Phys.* **1993**, *98*, 7029–7039.

(2) Schreiber, M.; Silva-Junior, M. R.; Sauer, S. P. A.; Thiel, W. Benchmarks for electronically excited states: CASPT2, CC2, CCSD, and CC3. *J. Chem. Phys.* **2008**, *128*, 134110.

(3) National Center for Biotechnology Information, PubChem Database. L-Proline. 2020; `https://pubchem.ncbi.nlm.nih.gov/compound/L-Proline`, CID=145742.

(4) Becke, A. D. Density-functional thermochemistry. III. The role of exact exchange. *J. Chem. Phys.* **1993**, *98*, 5648–52.

(5) Neese, F. The ORCA program system. *WIREs Computational Molecular Science* **2012**, *2*, 73–78.

(6) Neese, F. Software update: the ORCA program system, version 4.0. *WIREs Computational Molecular Science* **2018**, *8*, e1327.

(7) Weigend, F. Accurate Coulomb-fitting basis sets for H to Rn. *Phys. Chem. Chem. Phys.* **2006**, *8*, 1057–1065.
